# Complicated versus complexity: when an old woman and her daughter meet the health care system

**DOI:** 10.1186/s12905-020-01092-5

**Published:** 2020-10-12

**Authors:** Märta Sund Levander, Pia Tingström

**Affiliations:** Department of Nursing, Medical faculty, Linköping University, Linköping, Sweden

**Keywords:** Elderly, Complexity theory, Urinary tract infection, Case study

## Abstract

**Background:**

Detecting infection in frail elderly is a challenge due to lack of specific signs and symptoms. We highlight the complex situation when an elderly woman with urinary tract infection (UTI) and her daughter meet the highly qualified health care system. The aim was to describe and analyze the process when an elderly individual with an acute infection encounters the healthcare system.

**Methods:**

A descriptive, retrospective Single Case Study design with a qualitative approach was used. Data from interviews with the old women and her daughter, medical record data and different regulatory documents were gathered and analysed with a qualitative content analysis. In a second step, the results were interpreted with concepts from the complexity theory. Complexity theory has been used as a conceptual framework for analysis or a framework for interpretation. In this study we are using the theory for interpretation by comparing the results with the complexity theory, which is explored in the discussion.

**Results:**

The latent content analysis of the daughter’s story is interpreted as though she perceives the situation as causing a life crisis and a threat to her mother’s entire existence. The old women herself does not take part in what is happening, though after returning to home she is trying to understand her behaviour and what has happened. The health care tries different diagnoses and treatment according to standardized care plans without success. When urinary tract infection is finally diagnosed and treated successfully, the old women recovers quickly.

**Conclusion:**

The healthcare system should embrace the complexity in the encounter with an elderly individual. However, we found that the immediate reaction from the healthcare system is to handle the patients’ problem as complicated by complexity reduction. Shortcomings are that elderly patients with multiple disorders are difficult to evaluate and triage “correctly” for later placement in the appropriate continuum of care, although the findings of this case study also imply that with time the system instead took on an approach of absorption of complexity.

## Background

In this case study we highlight the complex situation when an elderly woman, in this paper with the pseudonyms Esther and her daughter, in this paper with the pseudonyms Maria, encountered the healthcare system. After a long and winding road in the healthcare system, Esther was finally diagnosed with urinary tract infection (UTI). We have earlier reported the challenge of detecting suspected infection in frail elderly people due to lack of specific signs and symptoms [1–4]. Clinically this leads to delayed diagnosis, worse outcome and risk of overuse of antibiotic treatment and hospital care. Nursing assistants (NAs) express suspicion of ongoing infection as “he/she is not as usual” [3, 4]. In this case Maria, Esther’s daughter, called for help using the same expression and thereby started the care process. When we came upon the case of Esther and Maria, we noticed how they described non-specific symptoms and related them to their feelings of not being listened to in the care process. We seek to explore what had happened and why, through a single case research study.

### Urinary tract infection in an elderly woman

Urinary tract infection (UTI) is the most common bacterial infection, accounting for approximately 20% of infections in community-dwelling elderly women [5, 6]. Despite several proposals by expert groups, a consensus on UTI guidelines does not exist [7]. Symptomatic UTI is defined by at least 2 of following criteria: fever (> 38 °C); frequency or urgency, dysuria, suprapubic tenderness, or costovertebral angle pain or tenderness; positive urine culture; and pyuria. Uncomplicated, symptomatic UTI is present when there is a symptomatic bladder infection indicated by urinary urgency or frequency, dysuria, suprapubic tenderness, costovertebral angle pain or tenderness with no recognised cause, and laboratory tests revealing UTI. However, a high prevalence of asymptomatic UTI, cognitive impairment, and comorbidity with advanced age, make diagnosis and management of a symptomatic UTI a challenge [8]. The spectrum of patient presentations of UTI varies from classic signs and symptoms to non-specific symptoms, such as increased lethargy, delirium, blunted fever response, and anorexia [7, 9]. Arinzon et al. [10] reported that post-menopausal women presented more severe and generalised non-specific symptoms, such as complaints of low abdominal pain, lower-back pain, constipation, and cold chills. As UTI is associated with increased morbidity, appropriate investigation and treatment are essential to decrease morbidity and avoid hospital care [11].

### The elderly patient and the healthcare system in Sweden

In Swedish healthcare three key criteria are used for setting priorities: *severity of the health condition; patient benefit; and cost-effectiveness.* These criteria are derived from the ethical principles established by the Swedish parliament [12]. Based on this ethical platform, emergency departments (EDs) categorise patients’ needs for care [13, 14]. In Sweden elderly people in need of daily support are taken care of by municipalities in the home or in special not-for-profit accommodation, e.g. nursing homes, or in private settings. Chronic diseases that require monitoring, treatment and daily care, and often life-long medication, place significant demands on the system [15]. General practitioners (GPs) collaborate with registered nurses (RNs) responsible for medical and nursing care, while NAs provide daily care [2, 3]. For all citizens, including the elderly, the healthcare organisation aim is for patients to visit the community healthcare centre for every-day healthcare needs and the ED for acute care. However, in a recent published governmental investigation [16] the Swedish healthcare system is described as focusing on hospital care rather than community-based healthcare. Patients turn to acute care at hospitals instead of close to home healthcare centres even for basic disorders and acute illnesses which actually could be handled at a less expensive level of care. One reason might be that community healthcare has office-hours and, for at least 20 years, there has been a constant lack of GPs, while EDs are open 24 h a day. Stiernstedt [16] concludes that the healthcare system in Sweden is of high quality but seems to be ineffective especially in encounters with elderly people with multimorbidity.

### The complexity theory

The healthcare system has often been described as a complicated system but instead it can be understood as a complex system according to the complexity theory [17–19]. The complexity theory deals with three (sometimes four) concepts: simple, complicated and complex problems with the four-part model including chaotic contexts [20]. Cilliers [21] describes an organisation and its complex system as being determined by the interactions of its components (i.e. individuals). The relationships are fundamental in the system and every organisation is unique. A complex system is characterised by uncertainty, unpredictability and interdependence among all members in a non-linear organisation, creating a system that is difficult to understand [19]. Zimmerman et al. [19] describe two ways of addressing this complexity: through complexity reduction or complexity absorption. Complexity reduction, in terms of standard procedures, guidelines, checklists, etc., might be the immediately desired striving to achieve clarity and focus. All in order to reduce the complexity in the situation to a complicated one, and possible to solve through guidelines etc. and use of linear cause and effect procedures. Complexity absorption refers to the degree to which organisations respond to their environment via more complex internal arrangements, and holds that effectiveness may be enhanced when internal organisational arrangements are designed to absorb complexity e.g. in decision-making processes [22]. Though this might not be the first choice, it can lead to increased understanding and learning. Begun, Zimmerman and Dooley [17] also emphasise that the complexity system is adaptive and that people within this system have the capacity to change and learn from experience.

Complexity theory has increasingly been incorporated within healthcare research [23], most commonly to conceptualise variables for data analysis or for interpreting findings. In this paper we are using this framework to analyse our single case on a micro level.

### Aim

To describe and analyse the process when an elderly individual with an acute infection encounters the healthcare system.

## Methods

Researchers used a descriptive, retrospective Single Case Study design (SCD) with a qualitative content analysis approach. SCD is often used to observe an individual (or a single unit) interacting with several variables [24]. Case studies allow collection of multiple sources of data and use of various analysis methods in order to understand deeply the complexity of the case. The data can be detailed, qualitative, quantitative or anecdotal, focusing on unique points of interest. Here, the case method is used to gather and analyse different types of data related to Esther’s and her daughter Maria’s experiences when encountering the healthcare deliverers, such as interview data, medical record-data and different regulatory documents.

### The case

The analysis used a relevance sampling method, also known as purposive sampling [25]. This means that all textual units that could contribute to answer our research questions were searched for and selected. The data collection started with interviews with the elderly woman Esther and her daughter Maria. With their permission, all medical record data were retrieved. Other text units such as regulatory documents were collected through websites and contact with healthcare professionals.

### Sample

Esther is a 90-year-old woman, living in senior housing for the elderly. She is on daily medication for constipation, thrombosis prevention, moisturising cream and, if necessary, painkiller Paracetamol 1000 mg and sleeping pills. She had a transient ischemic attack (TIA) 1 year earlier, and has vision loss and urine incontinence, otherwise she is essentially healthy with no cognitive decline. She needs a walker to support mobility and a little supervision to cope with personal hygiene. Esther’s daughter Maria is 46 years old, living in a nearby town. She often visits Esther for support.

### The healthcare context

The context is a not-for profit district county hospital with 200 beds and 7 primary healthcare centres, serving approximately 110,000 inhabitants in southern Sweden.

### Data collection

#### Interview data

The interviews with Esther and Maria were performed by one of the authors (MSL). Esther was interviewed in her home with her daughter Maria present, though Maria did not take an active part in the interview. Maria was interviewed at the interviewer’s home. The interviews lasted for a total of 2.5 h and generated 34 pages of written material (14,200 words). The interviews were transcribed verbatim by an independent transcriber. The accuracy of the transcription was checked by one of the authors (MSL) by comparing the transcription with the recorded interviews. The interviews aimed to encourage Esther and her daughter to tell their story of the events when Esther was diagnosed with UTI. An interview guide with open questions was developed and used as the basis for the interviews. After asking about background data the interview started with asking the respondent “Please, tell me when you / your mother became acutely ill this summer”. The respondent then told her story freely. Follow-up questions, such as “can you tell me more” and “what/how do you think?”, were used for clarification and to stimulate respondents to tell their stories in their own words.

#### Medical records

Esther’s patient records from community healthcare, primary health care, ED and hospital care were collected for a period of 5 months before and after the UTI event. The documentation comprised medical day notes, medical and nursing care summaries, and laboratory analysis. Documentation related to UTI was included in the content analysis.

### Regulatory documents

#### National guidelines

Textual data produced by National boards and clinics in the local hospital or community healthcare were searched and collected. We located Swedish Association of Local Authorities and Regions (SKR) [26], Public Health Authority [27], and Swedish collaboration to prevent antibiotic resistance (STRAMA) [28] as national sources concerning treatment of UTI which guide physicians in Sweden. Another source was “New guidelines for treating urinary tract infection in women” by André and Mölstad [29]. This document is published in the national journal *Läkartidningen* by The Swedish Medical Association (SMA, i.e. the union and professional organisation for physicians in Sweden; www.slf.se). In the guidelines the authors refer to the Swedish association for GPs (SFAM), which is associated with SMA and the Swedish Society of Medicine (SSM), the scientific organisation of the Swedish medical profession (www.sls.se). The International Statistical Classification of Diseases and Related Health Problems – Tenth Revision for use in Sweden (ICD-10-SE) [30] is frequently used in Esther’s medical record. When a diagnosis is agreed upon within the healthcare system a classification code is noted in the medical records. Both instructions for use of ICD-10-SE [30] and the codes have been used in the analysis.

At the studied ED the Rapid Emergency Triage and Treatment System (RETTS) was used [31, 32] Triage is an assessment of a patient’s medical severity based on history, symptoms and vital parameters to prioritise patient’s needs for medical examination. It is a short paper version of the original text book published in Swedish by Widgren [31]. The paper version is called “a short practical guide” version 1.0/2013. RETTS is published in Swedish, Danish and English. A Nordic steering group is updating the guidelines every year.

### Analysis

Qualitative content analysis is often used in health studies and focuses on the characteristics of language as communication, both regarding the content and/or the contextual meaning of the text [33]. In this case study we were interested in how different stakeholders express themselves and how they understand each other through verbal communication and in text-based documents (medical records, regulatory documents). We were also interested in the context in which the communication takes place and how it might have an impact on mutual understanding. The categories of expressions can describe both explicit and inferred communication. Several analyses of the material from different perspectives and with different “lenses” (Esther’s, Maria’s and the healthcare system’s) were performed to capture complex situations in instances where the individual interacts with several agents, such as with NAs, community healthcare RNs, pre-hospital staff, GPs, or staff at the ED, etc. Esther and her daughter Maria were not only interacting with staff as individuals; they also interacted with the healthcare system in the form of enacted regulatory documents. All these agents were used in different combinations. A qualitative content analysis according to Krippendorff’s analysis model was used [25]. Codes which categorised significant words and expressions to describe the care process were used to summarise notes from medical records, interviews and regulatory documents.

### Interpretation

Complexity theory has been used in a variety of ways in research [23] as a conceptual framework, a framework for analysis or a framework for interpretation. In this study we are using the theory for interpretation by comparing the results with the complexity theory, which is explored in the discussion.

## Results

### A journey through the healthcare system

**Fig. 1:**
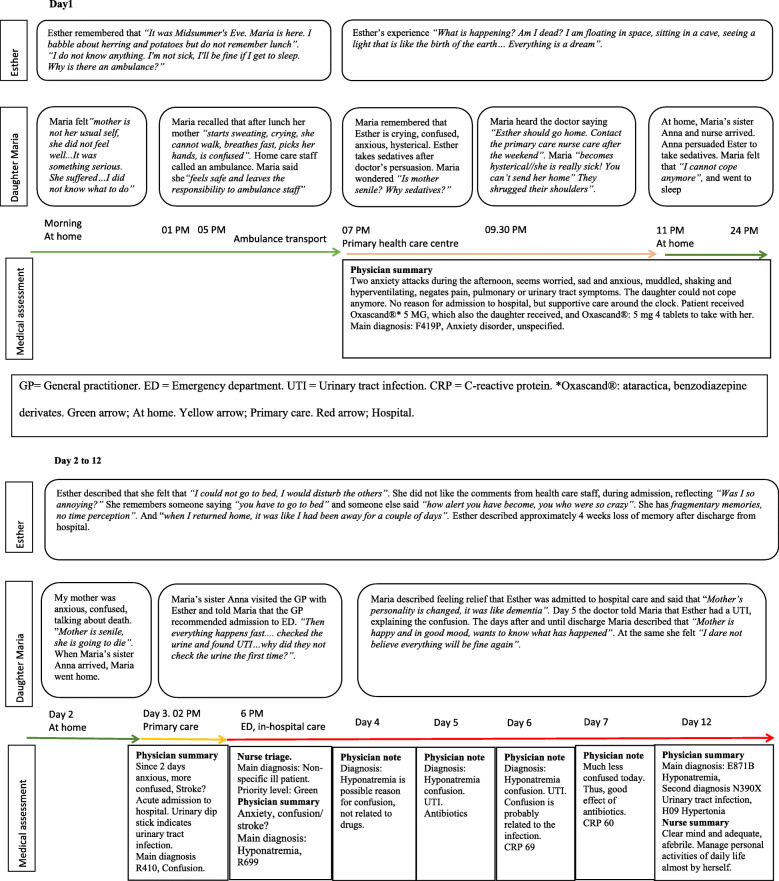
Extractions from interviews and medical files, illustrating the perspectives of Esther, her daughter Maria and medical assessment

Esther and Maria’s recounting of the UTI event extends over a significantly longer period than the time frame documented in the medical records. Maria described that her mother was not her usual self a long time before the UTI was diagnosed: *“There was probably something going on quite a long time before “/ ... ../“ [I] had no idea whatsoever about what it could be at all. It was the more general … supply, it couldn’t .. I didn’t get it”*. Also, Esther admits that she did not feel as usual, but that she did not want to bother anyone. *“I didn’t feel well, I felt it was something that wasn’t good. I will not nag [anyone] about it / …*. / *If the children heard this, they [would] just become worried./ … .../ I was not my usual self “.* Finally, Maria realised that her mother was seriously ill, and contacted home-care staff, who called for an ambulance. According to medical records the event took place over 12 days, including contact with different levels of healthcare. i.e. community care, primary care, emergency care and finally in-hospital care. Figure 1 illustrates the 12-day process by summarising Esther’s and Maria’s experiences, with notes from medical records. The timeline shows Esther and Maria’s journey through the healthcare system’s different care levels. The medical process to understand Esther’s illness, based on notes from GP/ED physicians and RN, comprises different diagnoses other than UTI, with a focus on hyponatremia and confusion. On day 5 Esther was diagnosed with UTI and at day 7 the notes in the medical records conclude that the antibiotics have had a good effect with less confusion.

### The encounter depends on expressions of signs and symptoms

Table 1 shows a summarised description of how the different agents, i.e. Maria, Esther, RETTS, medical record, national guidelines and ICD-10 SE) linguistically use expressions in the encounters. The summary comprises significant words and expressions from interviews, medical records and regulatory documents. Maria addresses her mother as a whole person, an individual that “is not her usual self”. She says that *“her personality is completely changed, like she was demented”*. Maria experienced her mother’s immediate need of care which she was trying to communicate verbally, as not being perceived as significant by the healthcare professionals. The latent content analysis of Maria’s story is interpreted as though she perceives the situation as causing a life crisis and a threat to her mother’s entire existence. In the encounters she experienced feelings of frustration, helplessness, apathy, fear, and doubt. She also expressed feelings of security, relief and acceptance when care professionals took over and freed her from responsibility. Esther herself did not take part in what was happening, she was in her own world. “*Next I was in space. / … … ../ And it was the earth so clear ... I thought it was fine”*. Her description also illuminates how she felt after returning from the hospital trying to understand her behaviour and what had happened.

**Table 1:** Signs and symptoms of UTI expressed by different agents; the patient, her daughter and the health-care system

The encounters between agents is enacted through expressions of signs and symptoms					
Daughter	Esther	RETTS	National guideline	ICD 10	Medical record
Mother was not her usual self, was not feeling well, was not OK, never been in such a condition, seemed to be feeling down, hysterical, sweating, crying, wailing, hyperventilating, seemed confused, picking with her fingers, seemed to disappear mentally, did not know where she is, not aware of what is happening. Her personality had completely changed, like she was demented.	I thought I was dead, I had no sense of time, I could not speak. It was like a cramp, I felt I was crazy	*Green*: Regularly checkups by nursing assistant, temperature 35 °C - 38.5 °C. Examination by physician within 240 min [13]	In women > 15 years of age New inconvenience of two of the following symptoms; smart, urgency and increased frequency of urination. Note: Non-specific symptoms, such as tiredness and confusion, should not be considered as UTI [29], SKR 2011 [26], STRAMA 2017 [28], Folkhälsomyndigheten 2019 [27].	F419P Unspecified anxiety R699 Unknown and unspecified causes of morbidity R410 Disorientated, unspecified confusion *Excludes* Psychogenic confusion (F44.8) [30]	Physical status: Normal, delayed reflex responses, good sleep. Cognitive status: Anxiety, confusion, picking with her hands, shaking, hyperventilate, disoriented, difficulty in finding words, babbles, do not follow instructions, cannot be alone. Biochemical markers: Normal, urine dipstick strong indication of UTI, CRP 69. Diagnosis: F419P. R 410 Confusion Unspecified anxiety, R 699, Hyponatremia, UTI [30]

*UTI* Urinary tract infection. The patient: 90-year-old Esther; the daughter; National guidelines; (International Classification of Diseases (ICD 10); Rapid emergency triage and treatment system (RETTS). Condensed extracts from data in the case is not time-dependent [13, 26–30]

According to RN triage and priority scheme RETTS, Esther is classified within the green category, i.e. “non-specific ill patient”. In the medical records Esther is reduced to a physical body, measurable with biochemical markers. Even her cognitive status is measured by predetermined criteria. National guidelines distinctly stress that non-specific symptoms, such as tiredness and confusion, should not be considered as UTI. Finally, the GP classified observations of Esther into ICD-10 SE codes such as R 410 and R 699, i.e. the classification system based on anatomy, the nature of the disease course and aetiology.

### Underlying regulations simplifies daily work

Table 2 summarises the guidelines in regulatory documents we have found to be applied in healthcare on a daily basis. The underlined meaning, i.e. the latent content, that emerges from these documents is illustrated in the theme *support aiming to simplify daily work for healthcare professionals.* This theme consists of the manifest categories *line up incoming patients, preventing antibiotic use for mankind,* and *ticking off for statistics.* To line up incoming patients RETTS suggest *prioritising need of car*e by *monitoring of vital signs*, such as respiration, pulse, blood pressure, and body temperature. The monitoring leads to five levels of priority*,* i.e. *colour coding according to severity in condition,* each of which involves different care. *Red* level means full monitoring of vital parameters, such as respiratory obstruction and rate, saturation, pulse, blood pressure, consciousness and seizure, with RN bedside and treatment until patient is assessed as at least level orange: time to medical examination 0 min. *Orange* level means full monitoring, as in *red* level, and body temperature > 41 °C or < 35 °C but with a 30 min interval and one RN responsible for the patient; time to medical examination < 20 min. *Yellow* level means selective monitoring, if necessary, of observed vital parameters and body temperature > 38.5 °C; time to medical examination < 120 min. *Green* level means no monitoring but regularly checkups of saturation, respiratory rate, pulse, alertness and body temperature 35 °C to body temperature 38.5 °C by NAs; time to medical examination < 240 min. *Blue* means no triage and that it is considered safe for the patient to wait for up to 4 h.

**Table 2:** Support system to simplify daily work for health-care professionals

	RETTS^**a**^	National guidelines	ICD-10-SE^**b**^
**Theme**	Support aiming to simplify daily work for health-care professionals		
**Category**	**Line up incoming patients**	**Prevent antibiotic use for humankind**	**Ticking off for statistics**
**Codes**	Prioritise need of car Monitoring vital signs Colour coding according to severity in condition	Identify patients at risk Correct diagnosis Correct treatment	Classification principles Full cover Manageability

^a^Rapid emergency triage and treatment system. ^b^ International Classification of Diseases

National guidelines: Swedish Association of Local Authorities and Regions (SKR [26]), The Swedish Medical Association (SMA) (André and Mölstad [29]), Swedish collaboration to prevent antibiotic resistance (STRAMA [28]), and Public Health Authority (Folkhälsomyndigheten [27])

The national guidelines for UTI focus on preventing antibiotic resistance by *identifying patients at risk* and documenting in the patient record a *correct diagnosis and treatment.* Patients at risk are individuals with impaired physical and cognitive ability due to disease or medical treatment such as neurological conditions or surgery. Correct diagnosis includes worsened urinary urgency or frequency, dysuria, and/or suprapubic tenderness, verified with urine culture. Correct treatment can mean treatment with antibiotics as well as no treatment. To prevent antibiotic resistance STRAMA gives recommendations for treating UTIs with antibiotics. The criteria are based on SMA, i.e. the presence of fever (> 38 °C), costovertebral angle pain or tenderness, genital complaints and at least 2 of following criteria: frequency or urgency, dysuria, suprapubic tenderness, positive urine culture; and pyuria.

The ICD-10 is produced by WHO as a basic classification for common medical statistical recording. It is “therefore not a list of “approved“ diseases nor nomenclature or a clinical terminology” [30]. Analysis of the introductory chapter in the Swedish instructions for classification revealed three principles, guiding how to use ICD-10: *classification principles*, meaning that the classification system occurs from anatomy, the nature of the disease course and aetiology. The aim of ICD-10 classify and describe statistically diseases and health problems. The second category, *full cover,* means that all imaginable disease conditions and health problems must be possible to code according to a functional system of classification. There is an awareness that this can be impossible at times and therefore “unspecified” is allowed. The third category, *manageability,* suggests that the classification system must be possible to handle for the user. Only diagnoses of relevance for care should be documented or if there are several diagnoses the most resource-demanding code should be used as a main diagnosis.

### Interpretation related to complexity theory

**Fig. 2:**
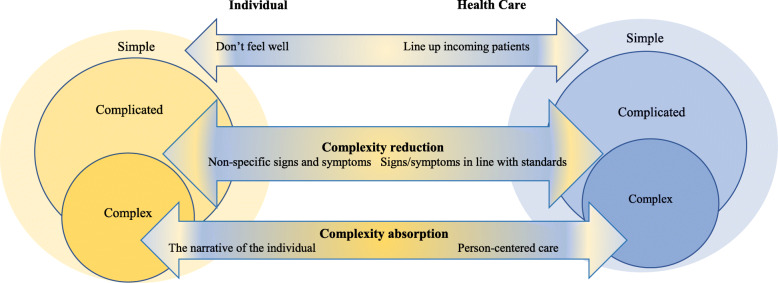
Model of the individual and healthcare encounters within the context of complexity theory

In this case, when Esther encountered the healthcare system she was handled as a patient with a complicated problem, i. e complexity reduction by assessing signs and symptoms in line with standards. Figure 2 summarises the findings as a model of the individual and healthcare encounters within the context of complexity theory. Ester and Maria turned to health care with, in their view, a clear and simple problem: Ester does not feel well, do something! Maria, as she was convinced that her mother was going to die, used strong expressions to emphasise her mother’s immediate need of care. When they arrived at the ED, a system to line up incoming patients, based on standard routines, was used, e.g. RETTS and ICD-10 SE (Tables 1 and 2). This could illustrate how the system uses complexity reduction in order to handle patients with different kinds of problems on a complicated level, i. e standardised procedures and routines. However, this system is not adapted to elderly persons with multimorbidity’. Esther at first received a classification of “green” (Fig. 1, ED day 3). Further on, according to the ICD-10 system she was classified with confusion and hyponatremia, which could be seen an attempt to state a diagnosis in line with her condition. This is also an example of how the health care system uses standard routines and guidelines to reduce uncertainty, unpredictability and interdependence in a complex situation, i.e. complexity reduction. Later in the care process (Fig. 1, day 6) the picture had changed. Staff seemed to keep two systems in their mind: the standardised care plans and a person-centred tailored care. The findings indicate that Maria’s description had been listened to and more signs and symptoms had been acknowledged. An alternative diagnosis was made: UTI which was successfully resolved with antibiotic treatment. This can be interpreted as an indication that the healthcare system can handle a problem with all its complexity and has the capacity to alter and learn from experience, i.e. complexity absorption. This means that the system can adapt and cope with uncertainty, unpredictability and interdependence. When Ester was treated according to her actual health problem (Fig. 1, day 7) her health improved.

## Discussion

This study gives insight into an elderly patient’s encounter with the complex healthcare system. The results can be interpreted to show that when complexity is not recognised, but handled as a complicated situation, the encounter results in misunderstanding, misjudgement, delayed diagnosis and unnecessary suffering. However, the findings imply that although the first reaction from the healthcare system was to take on complexity with reduction, with time (Esther was hospitalised) an opportunity arose to take on absorption of complexity.

In the clinical context, complexity absorption can be seen as being in line with the concept of person-centred care emphasising the encounter as interactions between two human beings, and not just a clinical meeting between a patient and a health-care provider [34]. In the clinical encounter the patient’s narrative is the core for seeing the patient as an individual with his or her personal identity [35]. The American Geriatric Society emphasises that the relationship between professional and patient is underpinned by central values such as mutual respect and understanding of the patient’s self-esteem and will [36]. The partnership formed through the person-centred meeting can lead to a joint understanding of how the present situation is experienced and the needs and resources available.

Any model of an organisation or an organisational system is an approximation and a simplification of reality. Models shape the way people believe the system works, and constrain or affect the way people thinks and the expectations the individual has [17]. In addition, the complex system has an inherent paradox: all actors in the encounters are part of different complex systems that might result in different views of reality. Hence, healthcare can be understood as the results of interactions between people [19]. In this case it means that Maria and Esther, as well as the healthcare system, have their own reasons to simplify reality when they communicate how to handle the problem. Maria communicated Esther’s needs for care due to her personal understanding of Esther’s condition as not being her usual self, expecting healthcare staff to act on this simple information. While on the other hand, the healthcare system is concerned with reduction of complexity, i. e making problems complicated and easier to solve through measurable, objective signs and manifestations based on pathophysiological facts.

The support systems for care in a Swedish context may seem to be contradictory. On one hand, the Swedish government emphasises that priorities of individual patients should never be set only by following predefined templates or criteria. Each case is unique and must be judged by the individual patient’s needs and the unique conditions in that particular situation, with guidance of thoughtful ethical principles [12]. Also, the Swedish National Board emphasises the importance of the initial priority of patients for safe emergency care and that the aim of triage is to improve patient safety [37]. The intention is to provide a guarantee of compliance with the parliament’s guidelines on priority setting – that patients with the greatest need are first in line to receive care. On the other hand, the intention with the classification system ICD-10- SE is strictly statistical, though in practice it is also used for purposes other than those intended: for control of quality of care, for business description and for resource allocations and search systems in the medical record [30], i.e. criteria for diagnosis.

In complex clinical practice with high standards of efficiency, it might be easier to rely on checklists and guidelines than adjust to individual perceptions. It could be seen as a way to make the complex situation fit into a complicated system where it is possible to specify standardised ways to handle unique situations and get the same result every time. RETTS is an example of complexity reduction that may be seen a prudent and appropriate approach to achieve practical guidelines for healthcare professionals [19]. However, the reduction has its limits, and might have a negative impact in terms of clarity, focus and utility [19]. In delivery of healthcare, shortcomings are that elderly people with multiple disorders are difficult to evaluate and triage “correctly” for later placement in the appropriate continuum of care. Healthcare professionals working in EDs apply systems for prioritising patients which might contribute to increased risk of mis-judgment, ending up in a lower triage level, particularly for elderly patients [37]. Frail elderly patients without specific symptoms are at particular risk of delayed or inadequate risk assessment with a risk of worsening outcome [13, 38]. Arvidsson et al. [39] also found that both GPs and RNs in primary care expressed a simplified interpretation of severity of illness and benefitted from ranked priority for medical examination of elderly patients. One example of such simplification was that many RNs based their ranking of severity of a condition on how imperative it was for the patient to see a physician. A common reflection was that the perceptions about severity of illness could differ from the perspective of the patient and staff, an insight that is obvious in the Esther case. Hence, complexity reduction is not able to capture fully a complex reality and sometimes requires assessments that go beyond the method; as all structured methods will not fit all actual cases. According to complexity theory [17–19] it appears that RETTS and ICD classifications would provide a better basis for assessment by combining the vital signs with the patient’s more subjective perspective, i.e. using complexity absorption instead of complexity reduction, which is confirmed in the case of Esther.

Though a single case study can provide valuable knowledge on complex situations and events, there are limitations to be aware of. It is necessary to be careful when discussing relationship between investigated variables within the case. SCD has no systematic data collection and the subjectivity in choosing data to include is obvious. In the traditional case study, there is no stated dependent variable and therefore conclusions should be drawn with caution. The strength of this study are the multiple sources and detailed interviews. This case study can contribute to in-depth insight about how to understand and handle the encounter between elderly patients and the healthcare system.

### Clinical implications

This case study emphasizes how healthcare have the potential to adjust and change from complexity reduction to complexity absorption. If healthcare is prepared to listen to and take in the narrative of the individual at the first encounter, it may provide a better basis for adequate diagnosis and treatment. A person-centered care might also shorten the care period.

## Conclusions

The healthcare system should embrace the complexity in the encounter with an elderly individual. However, we found that the immediate reaction from the healthcare system is to handle the patients’ problem as complicated by using complexity reduction. Shortcomings are that elderly patients with multiple disorders are difficult to evaluate and triage “correctly” for later placement in the appropriate continuum of care, although the findings of this case study also imply that with time the system instead took on an approach of absorption of complexity.

## Supplementary information

**Additional file 1.** Interview guide

## Data Availability

The datasets generated and/or analysed from interviews with the respondents during the current study are not publicly available due to confidentiality but are available in Swedish from the corresponding author on reasonable request. All data generated or analysed from official documents during this study are included in this published article with links in the reference list.

## Abbreviations

ED: Emergency department
GP: General Practitioner
ICD 10-SE: International Statistical Classification of Diseases and Related Health Problems – Tenth Revision for use in Sweden
NA: Nursing assistants
RETSS: Rapid Emergency Triage and Treatment System
SFAM: The Swedish Association of General Practice
RN: Registered Nurse
SCD: Singe Case Design
SKR: Swedish Association of Local Authorities and Regions
SMA: Swedish Medical Association
SSM: Swedish Society of Medicine
STRAMA: Swedish collaboration to prevent antibiotic resistance
UTI: Urinary tract infection

## References

[1] Sund Levander M, Grodzinsky E. The challenge of infections in frail elderly: The story of Mr Nilsson. Clin Med Rev Case Rep. 2015:2(9):1–2. Online publication.

[2] Sund-Levander M, Tingstrom P (2013). Clinical decision-making process for early nonspecific signs of infection in institutionalised elderly persons: experience of nursing assistants. Scand J Caring Sci.

[3] Tingström P, Milberg A, Rodhe N, Grodzinsky E, Sund-Levander M (2015). Nursing assistants: “he seems to be ill”– a reason for nurses to take action: validation of the early detection scale of infection (EDIS).

[4] Tingström P, Milberg A, Sund-Levander M (2010). Early nonspecific signs and symptoms of infection in institutionalized elderly persons: perceptions of nursing assistants. Scandinavian J Nurs Sci.

[5] Berman P, Hogan D, Fox R (1984). The atypical presentation of infection in old age. Age Ageing.

[6] Kovach C, Logan B, Simpson M, Reynolds S (2010). Factors associated with time to identify physical problems of nursing home residents with dementia. Am J Alzheimers Dis Other Dement.

[7] Detweiler K, Mayers D, Fletcher SG (2015). Bacteruria and urinary tract infections in the elderly. Urol Clin North Am.

[8] Mody L, Juthani-Mehta M (2014). Urinary tract infections in OlderWomen. A clinical review. JAMA Clin Rev Educ.

[9] Matthews SJ, Lancaster JW (2011). Review article: urinary tract infections in the elderly population. Am J Geriatr Pharmacother.

[10] Arinzon Z, Ahabat S, Peisakh A, Berner Y (2012). Clinical presentation of urinary tract infection (UTI) differs with aging in women. Arch Gerontol Geriatr.

[11] Robinson D, Giarenis I, Cardozo L (2015). The management of urinary tract infections in octogenerian women. Maturitas..

[12] Priorities in health care (1996). Proposition 1996/97: 60 (Prioriteringar inom hälso- och sjukvården. Regeringens proposition 1996/97:60; PROP 1996/97:60) 1996/97:60.

[13] Sandman K, Ekerstad L, Indroth N (2012). Triage som prioriteringsinstrument på akutmottagning – en etisk analys av RETTS (Triage as priority instruments in the emergency room - an ethical analysis of Rett).

[14] SBU (2010). Triage and flow processes at the emergency room. A systematic literature review. Swedish. Triage och flödesprocesser på akutmottagningen. En systematisk litteraturöversikt.

[15] SI. Health care in Sweden: Swedish Institute; 2017. https://sweden.se/.

[16] Stiernstedt G (2016). Effektiv vård (Effective Care) Slutbetänkande.

[17] Begun J, Zimmermann B, Mick SWM (2003). Health care organization as complex adaptive systems. Advances in health care organization theory.

[18] Glauberman S, Zimmerman B. Complicated and complex systems: what would successful reform of medicare look like?: Commission on the Future of Health Care in Canada: Commission on the Future of Health Care in Canada. York: York University; 2001.

[19] Zimmerman B, Dubois N, Houle J, Lloyd S, Mercier C, Brousselle A (2011). How does complexity impact evaluation? An introduction to the special issue. Canadian J Program Eval.

[20] Schloss E (2014). A dynamic framework for planning under simple, complicated, and complex conditions. Emergence Complex Org.

[21] Cilliers P (2000). What can we learn from a theory of complexity?. Emergence..

[22] Waltera B, Bhuian S (2004). Complexity absorption and performance: a structural analysis of acute-care hospitals. J Manag.

[23] Thompson DS, Fazio X, Kustra E, Patrick L, Stanley D (2016). Scoping review of complexity theory in health services research. BMC Health Serv Res.

[24] Nock MK, Michel BD, Photos VI (2008). Single-case research designs. In: Mckay D, editor. Handbook of research methods in abnormal and clinical psychology housand.

[25] Krippendorff K (2013). Content analysis. An introduction to its methodology.

[26] SKR (2011). National initiative for increased patient safety. Health-related urinary tract infections; Preventive measures (Nationell satsning för ökad patientsäkerhet. Vårdrelaterade urinvägsinfektioner; åtgärder för att förebygga). Swedish.

[27] Folkhälsomyndigheten (2019). Behandlingsrekommendationer för vanliga infektioner i öppenvård (Recommendations for usual infections in primary care). Swedish.

[28] STRAMA (2017). Läkemedelsbehandling av urinvägsinfektioner öppenvård – behandlingsrekommendation (Treatnment of utinary tract uinfection in primary care . recommendations). Swwdis. STRAMA (Samverkan mot antibiotika resistans).

[29] André M, Mölstad S (2008). New guidelines for treating urinary tract infection in women (in Swedish). (Nya riktlinger för urinvägsinfektion hos kvinnor). Läkartidningen..

[30] ICD10-SE International Statistical Classification of Diseases and Related Health Problems, Tenth Revision (ICD-10) (2018). Internationell statistisk klassifikation av sjukdomar och relaterade hälsoproblem – Systematisk förteckning, svensk version 2018. ICD-10-SE.

[31] Widgren BR (2012). RETTS : Akutsjukvård direkt.

[32] Widgren BR, Jourak M (2011). Original contribution: medical emergency triage and treatment system (METTS): a new protocol in primary triage and secondary priority decision in emergency medicine. J Emerg Med.

[33] Hsieh H, Shannon S (2005). Three approaches to qualitative content analysis. Qual Health Res.

[34] Ekman I (2014). Editor. Personcentrering inom hälso- och sjukvård. Från filosofi till praktik (person centering in health care. From philosophy to practice) in Swedish.

[35] Ekman I, Swendberg K, Taft C, Lindseth A, Norberg A, Bring E (2011). Person-centered care-ready for prime time. Eur J Cardiovasc Nurs.

[36] Care. AGSEPoPC (2016). Person-Centered Care: A Definition and Essential Elements. J Am Geriatr Soc.

[37] Hsia RY, Wang E, Saynina O, Wise P, Pérez-Stable EJ, Auerbach A (2011). Factors associated with trauma center use for elderly patients with trauma: a statewide analysis, 1999–2008.

[38] Rutschmann O, Chevalley T, Zumwald C, Luthy C, Vermeulen B, Sarasin F (2005). Pitfalls in the emergency department triage of frail elderly patients without specific complaints. Swiss Med Weekly.

[39] Arvidsson E, André M, Borgquist L, Carlsson P, Lindström K (2007). Så resonerar läkare och sjuksköterskor vid prioriteringar av patienter i primärvård (this is how doctors and nurses reason for the priorities of patients in primary care). Swedish.

