# Supplementary material for: Complicated versus complexity: when an old woman and her daughter meet the health care system
Source: BMC Womens Health. 2020 Oct 12;20:230. doi: 10.1186/s12905-020-01092-5 (PMC7552441; doi:10.1186/s12905-020-01092-5)
Supplement: Supplementary file 1 — Additional file 1. Interview guide [file 12905_2020_1092_MOESM1_ESM.docx]

**Interview guide**

Background data

- Age, Social, family, living condition, earlier profession
- Esther's need for help from home care

Introductory question

Please tell me about when you / your mother became acutely ill this summer.

During the interview, the interviewer asked questions in order to deepen the story, such as

"How do you think then?", "Can you describe it more?" etc.

The interview ended with the respondent being given the opportunity to reflect and give views on the situation to be interviewed.
